# Rescue from liver transplantation: TIPSS and thrombectomy successfully treat a case of acute Budd–Chiari syndrome complicated by portal vein thrombosis

**DOI:** 10.1259/bjrcr.20160059

**Published:** 2016-07-27

**Authors:** Sarah A Townsend, Salil Karkhanis, Dhiraj Tripathi, Paolo Mueisan, Zergham Zia, Ahmed M Elsharkawy

**Affiliations:** ^1^Liver Unit, Queen Elizabeth Hospital, Birmingham, UK; ^2^Radiology Department, Queen Elizabeth Hospital, Birmingham, UK

## Abstract

We report the rare case of a female who presented with fulminant liver failure secondary to acute Budd–Chiari syndrome and complete portal vein thrombosis. She met the criterion for liver transplant and was transferred to our care for assessment and further management. Transplant was deemed a too-high risk and so rescue therapy was undertaken using mechanical thrombectomy and transjugular intrahepatic portosystemic shunt insertion to decompress the portal system. The patient made a full recovery. This is a rare case report of a patient meeting liver transplant criteria secondary to acute Budd–Chiari syndrome and complete portal vein thrombosis, which was managed successfully entirely by radiological means; this technique could be used to avoid or act as a bridge to liver transplantation in the future.

## Introduction

Budd–Chiari syndrome (BCS) is a rare disorder caused by hepatic venous outflow obstruction at any level from small hepatic veins (HVs) to the junction of the inferior vena cava and the right atrium. The presentation can be either acute or chronic, with presenting features varying from non-specific mild abdominal pain to fulminant liver failure. Its sequelae can progress rapidly over weeks to months.^1^ One possibility is that of acute or chronic portal vein thrombosis (PVT), which is reported concomitantly in BCS in 15–25% of cases.^2^ Evidence for treatment of this complicated pathology is limited to case reports and series at best. With such ambiguity, deciding on the best treatment strategy for an individual patient can be difficult.

BCS occurring in isolation has traditionally been managed in a stepwise manner with anticoagulation and medical therapy in the first instance. In the case of non-response or visible stenosis, this can be followed by angioplasty/stenting or transjugular intrahepatic portosystemic shunt (TIPSS).^2,3^ Surgical portosystemic shunting has traditionally been the next step for patients in whom medical therapy has failed or where there are no radiological options, and some studies have demonstrated impressive results.^4^ However, while radiological expertise in these cases continues to grow, surgical experience is diminishing, and surgical options have generally been superseded by radiological methods.^3^ Liver transplantation (LT) is generally reserved as rescue therapy in the event of poor response to TIPSS.^3^

Isolated portal vein and mesenteric vein thrombosis (PVMVT) is managed with a completely different approach. Initial management is always with systemic anticoagulation and medical therapy. In case of non-response, the current trend is to proceed to endovascular clot removal or dissolution [transhepatic portal thrombectomy/thrombolysis and/or superior mesenteric artery catheter-directed thrombolysis (CDT)].^5–7^ We discuss a case of BCS with extensive acute PVMVT, who presented to our unit with fulminant liver failure and was managed entirely by a radiological approach.

## Clinical presentation

A 63-year-old female patient was referred to a neighbouring tertiary liver centre, having presented to her local hospital with a 9-day history of abdominal pain, nausea and vomiting. Clinical examination revealed ascites and jaundiced sclera, but was otherwise unremarkable for stigmata of liver disease. She had a past medical history of chronic obstructive airways disease and hypothyroidism, and a recent alcohol intake of 50 units per week following a family bereavement, but no history of chronic liver disease and no long-term alcohol dependence.

## Investigations

**Figure 1.**
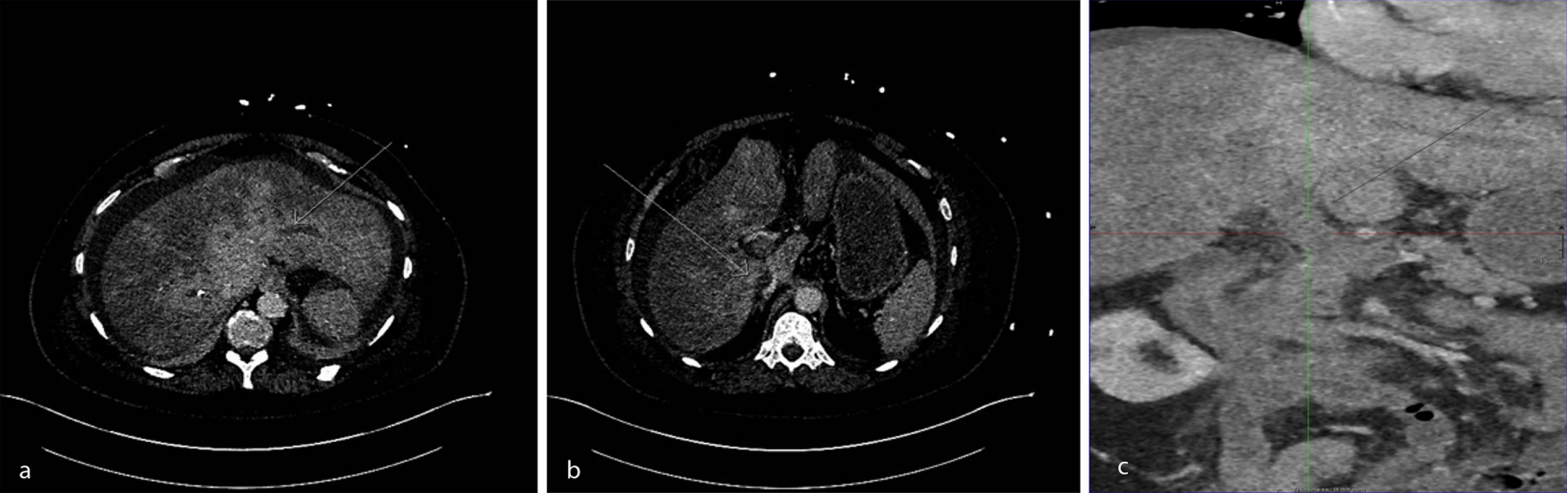
Pre-procedure contrast-enhanced CT scan of the abdomen in the portal venous phase demonstrating occlusive thrombus in the (a) hepatic veins (arrow), (b) inferior vena cava (arrow) and (c) main portal vein (arrow), extending into the splenic vein and superior mesenteric vein. All the images demonstrate a background hepatic parenchymal congestion with a mosaic enhancement pattern, giving a “nutmeg” appearance.

Abnormal liver biochemistry and coagulopathy were identified on initial serum investigations (Table 1), and a CT scan of the thorax, abdomen and pelvis was performed. This showed extensive filling defects in the hepatic venous confluence, HVs, PV and superior mesenteric vein (SMV), and a patent splenic vein (SV), which was consistent with acute thrombotic disease (Figure 1a–c). No underlying malignancy or background cirrhosis was identified. Based on these findings, a diagnosis of BCS complicated by PVMVT was made.

**Table 1.** Serial blood test results

Blood test parameters	Admission	2 days post-intervention	5 months	8 months
Haemoglobin (g l^−1^)	112	82	113	120
White blood cells (10^9^ l^−1^)	10.7	15.0	7.2	6.8
Platelets (10^9^ l^−1^)	127	150	238	208
International normalized ratio	1.7	1.5	2.2	2.7
Alaninaminotransferase (U l^−1^)	779	167	32	30
Alkaline phosphatase (U l^−1^)	214	153	379	349
Total bilirubin (μmol l^−1^)	80	295	30	30
Albumin (g l^−1^)	34	25	45	45
Sodium (mol l^−1^)	135	137	147	4.4
Creatinine (mol l^−1^)	102	164	127	111

As the presentation suggested an acute thrombosis, a decision was made at the presenting hospital to pursue CDT, and this was commenced on day 1. Subsequent daily venograms showed no significant change in the size of the thrombus. On day 3, the patient developed hepatic encephalopathy and was transferred to our centre, the regional liver transplant unit, for transplant assessment.

Following transfer, it was confirmed that she met the listing criteria for super-urgent LT under category 7 of the UK Liver Transplant Policy.^8^ However, concerns were raised by the surgical team regarding the patient's age and comorbidities, and the feasibility of LT in view of extensive inflow and outflow thrombus. In-depth multidisciplinary review of the case concluded that an endovascular approach was likely to provide the best outcome.

## Treatment

On day 5, the patient underwent platelet and fresh frozen plasma transfusion in preparation for the planned TIPSS procedure. The procedure was performed under general anaesthesia in the angiography suite with the patient in the supine position. The pre-existing right internal jugular vein thrombolysis catheter was removed over a wire and access secured with a 10 French standard vascular sheath. Multiple attempts were made to pass the TIPSS needle from the HV to the PV using a Cook^® ^Rösch-Uchida transjugular access kit (Cook Medical, Bloomington, IL). A combination of fluoroscopy and transabdominal ultrasound was used to direct the PV entry and confirm position. Wire and catheter were first navigated into the main PV and then into the SMV and SV, which confirmed the presence of extensive occlusive thrombus. The SV was observed to be patent with sluggish flow.

**Figure 2.**
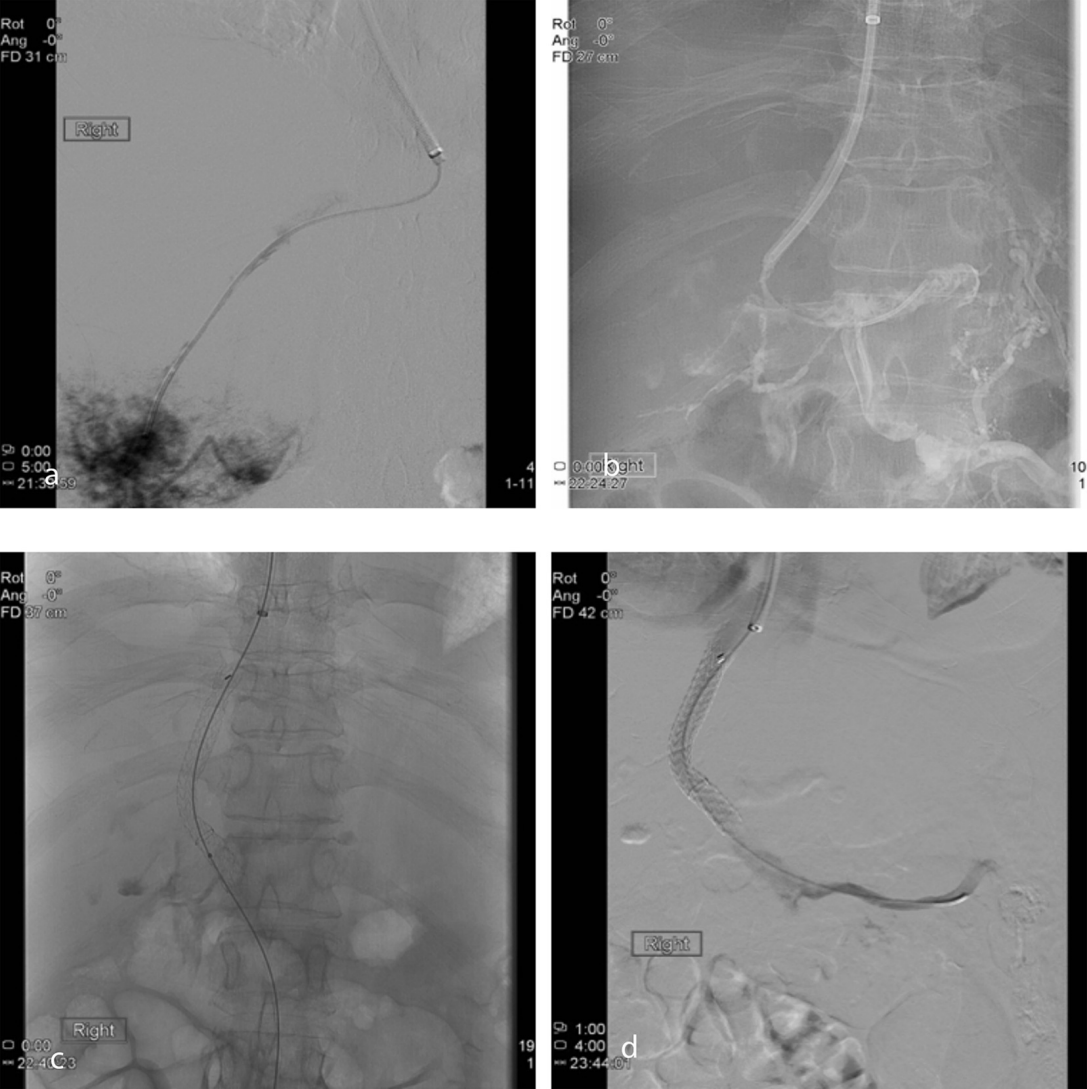
Images taken during the procedure demonstrating initial hepatic wedge venogram with filling defects within an occluded hepatic vein (a). Puncture of right PV and venogram demonstrated extensive occlusive thrombus in splenic vein, superior mesenteric vein, and main PV, confirming the CT scan findings (b). Following tract dilatation, a transjugular intrahepatic portosystemic shunt stent graft was successfully placed (c), with the venogram showing good flow through the stent graft but non-occlusive thrombus in the main PV (d). PV, portal vein.

After securing access with a guide wire and predilating the tract with a standard 8 mm balloon, a GORE^®^ VIATORR 10 mm × 7 cm stent graft (W. L. Gore, Flagstaff, AZ) was placed. Post-dilatation was performed with a standard 10 mm balloon. Once the outflow was secured, the patient was anticoagulated with a bolus of 5000 units of intravenous heparin. The Angiojet™ Ultra Peripheral and Solent Proxi Thrombectomy system (Bayer Medical Care Inc., Indianola, PA) was then used to pulse-spray 10 mg of Actilyse^®^ (recombinant tissue plasminogen activator; Boehringer Ingelheim Ltd, Bracknell, UK) into the PV and SMV thrombus, followed by mechanical thrombectomy (MT). Good recanalization and hepatopetal flow was established in the PV, SMV and SV, with minor residual non-occlusive thrombus in the SMV (Figure 2a–d). After TIPSS, pressure measurements demonstrated a PV pressure of 29 mmHg and right atrial pressure of 24 mmHg, giving a gradient of 5 mmHg. A Cragg-McNamara^®^ valved infusion catheter (Microtherapeutics Inc., Irvine, CA) was used to inject an additional 3 mg of Actilyse. At this point, the patient was noted to have epistaxis and haematuria. A decision was therefore made to not proceed with overnight catheter thrombolysis as previously planned. The 10 French vascular sheath was left *in situ* for future access and the patient was transferred back to the intensive care unit, where she remained stable. Fibrinogen levels were checked and found to be within normal range at 1.7 g l^−1^, and so low-molecular-weight heparin was continued.

On day 7, the patient was recalled to the angiography suite for a follow-up TIPSS venogram, which demonstrated a patent TIPSS stent and SV. There was also a short segment occlusive thrombus in the SMV with collateralization. Initial aspiration thrombectomy with an 8 French catheter was unsuccessful. Subsequently, cautious pulse-spray thrombolysis with 5 mg of Actilyse in combination with MT was carried out. Only a small volume of residual non-occlusive SMV thrombus remained at the end of the procedure. Intravenous heparinization was continued overnight. A favourable TIPSS pressure gradient of 4 mmHg was measured.

**Figure 3.**
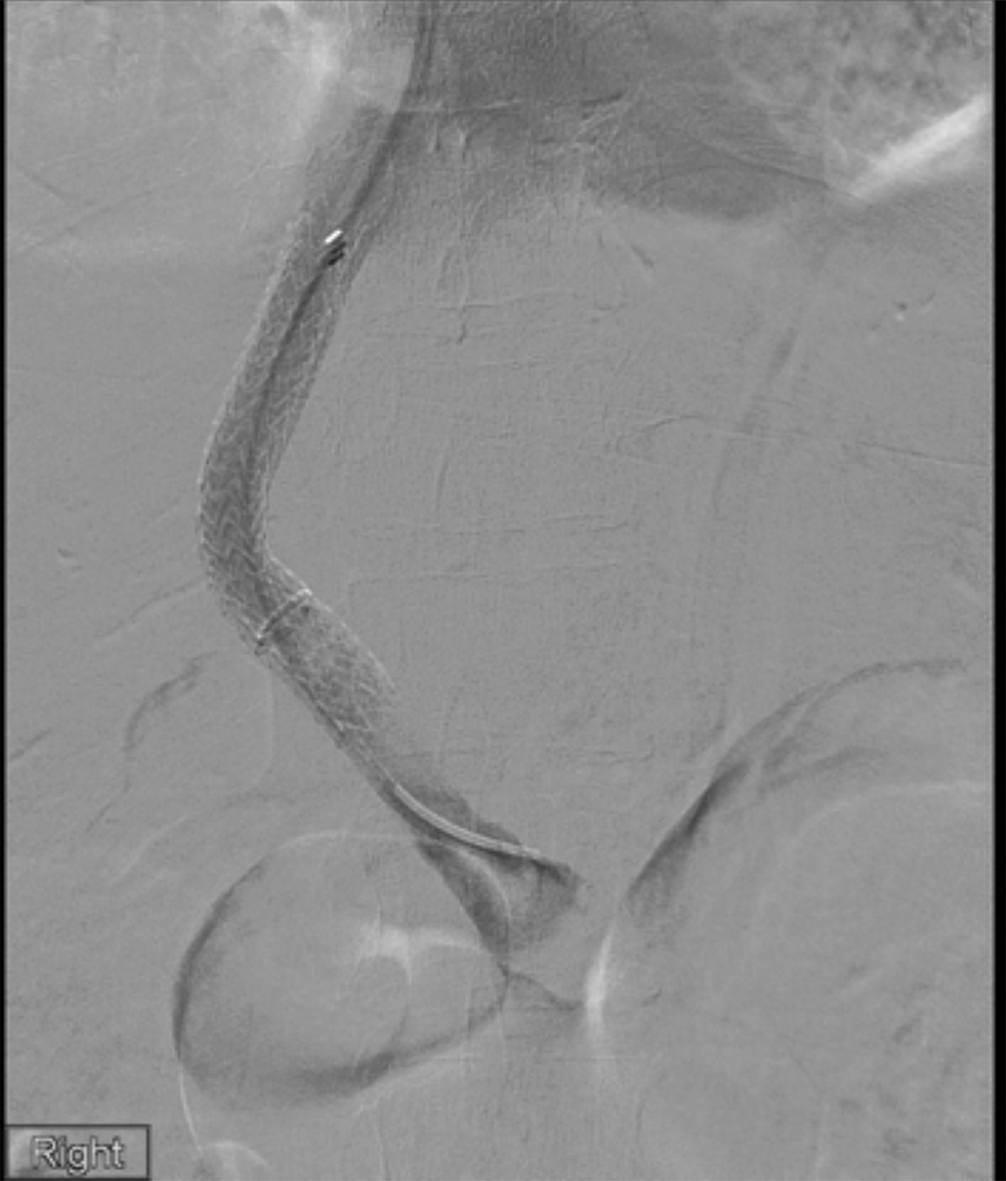
Follow-up venogram 3 days post-procedure, following sessions of thrombolysis and pharmacomechanical thrombectomy, demonstrated minimal non-occlusive main portal vein thrombus and good flow through the stent graft, which was deemed acceptable at the time.

A repeat TIPSS venogram on day 8 (Figure 3) demonstrated persistent small volume non-occlusive thrombus in the SMV, which was macerated with an 8 mm balloon. Subsequently, a bolus of 3 mg of Actilyse was injected into the thrombus, followed by continuous infusion at 0.5 mg/h for 6 h. A final venogram on day 8 confirmed minimal residual thrombus in the SMV, and a decision was made that no further procedures were required. The internal jugular vein sheath was removed with manual haemostasis.

Although recovery was complicated by renal impairment and pulmonary oedema, which required haemodialysis, the patient was later discharged with both renal and liver function returning to baseline (Table 1) and no ascites, on only oral anticoagulation therapy.

Outpatient follow-up with a combination of CT and ultrasound of the liver up to 1 year post--procedure demonstrated a patent TIPSS stent with hepatopetal flow in the PV. The patient remains well, and screening tests for malignancy, prothrombotic and myeloproliferative disorders have all been negative.

## Discussion

BCS is a complex and rare pathology, having been reported in 0.05–0.5% of autopsy studies,^9^ which limits the available evidence for management guidelines to case series and retrospective studies. This makes it difficult to reach a verdict on the best management plan. Even rarer is the presence of PVMVT complicating BCS, and the available evidence is yet more sparse and limited to case reports and a single case series. Prognosis for these patients is thought to be worse than for BCS alone, with mortality rates as high as 70%,^5^ and limited treatment options.

The primary goal of BCS treatment is reduction of hepatic congestion and associated sequelae. The aetiology of BCS also plays a key role in planning appropriate therapy. In this case, extensive investigation did not reveal any risk factors (prothrombotic states, malignancy, etc.), which has been found to be the case in as much as 84% of cases in a recent prospective, multicentre study.^10^ Medical management includes control of underlying disease and lifelong anticoagulation.^11^ There is some evidence for both systemic thrombolysis and CDT, with possible superiority of CDT in patients with acute thrombosis.^12^ However, studies have demonstrated that thrombolysis alone is not always successful, and angioplasty and/or stenting may be required if a focal underlying stenosis is demonstrated.^13^ While LT has been shown to be the treatment modality offering maximum longevity, donor organs are not always forthcoming; therefore LT for BCS is reserved for those with an acute presentation and fulminant liver failure. In such cases, TIPSS can prove to be a valuable “bridge” to transplantation,^8^ and may be superior to recanalization of the HV alone. Tripathi et al^14^ published long-term results of 67 BCS patients treated with TIPSS and demonstrated the efficacy of symptom control in 97% of patients, with 99% secondary patency and 10-year survival of 72%. The study recommended TIPSS as a first-line, and possibly definitive, management technique. Surgical methods such as portocaval and mesocaval shunts are no longer widely used.^14^

Comparatively, evidence concerning isolated PVMVT is greater, but is still limited to case series and retrospective reviews. Medical management is recommended in the first instance, including systemic anticoagulation. A prospective, multicentre study demonstrated a recanalization rate between 38% and 40% using heparin followed by oral vitamin K antagonists.^5^ CDT is performed *via* the superior meseteric artery, transhepatic or transjugular routesin cases of failure of anticoagulation. A review by Hall et al^15^ demonstrated 40% complete and 45% partial recanalization, with an overall bleeding complication rate of 60%. There have been studies combining this with MT for rapid clearance of thrombus with reasonably good results.^15–17^ In a case described by Sze et al^17^ in 2000, a patient with acute complete SMV thrombosis and partially occlusive PV thrombus was treated successfully by pharmacomechanical thrombolysis, following failed intra-arterial thrombolysis. Flow was re-established in the PV and SMV using mechanical aspiration of the thrombus followed by balloon angioplasty and stent insertion.^18^ Surgical thrombectomy is reserved for cases demonstrating symptomatic end organ dysfunction such as bowel ischaemia, where a laparotomy can be combined with thrombectomy.

The combined presence of BCS and PVMVT is rare, with no definite agreed algorithm for management. Published cases generally seem to follow a combination of management principles of the above two pathologies. Those reported used similar techniques with the addition of TIPSS insertion to facilitate decompression of the portal system and create an adequate hepatic outflow tract.^12,19,20^ A review by Uflacker^20^ in 2003 recommended that TIPSS should be attempted in all patients with acute PVT who have underlying cirrhosis in order to reduce the risk of recurrent thrombosis secondary to poor blood flow and stasis. Subsequent case reports of patients with acute PVT and BCS have demonstrated the successful use of TIPSS in combination with mechanical thrombolysis, usually following failed anticoagulation or thrombolysis.^2,21,22^ A slightly different approach of anticoagulation first, followed by TIPSS, was seen in a large series of 204 BCS patients, of which 18 had associated PVMVT. Survival was worse in BCS and PVMVT than in isolated BCS.^23^

In cases reported previously, the PV thrombosis described was incomplete and none of the patients met transplant criteria. Our patient had complete PV thrombosis, which made it a challenging TIPSS case. While PVMVT is not a contraindication for LT, it is an independent prognostic marker for post-LT survival, and increases the risk of re-thrombosis and re-transplantation.^24^ Added to this were the concerns around the patient’s ability to cope with the physiological stress of LT given her age and comorbidities. The endovascular option was therefore chosen as a rescue modality. CDT had failed, most likely, owing to poor inflow from PV thrombosis. In cases of BCS where there is concurrent PVT, TIPSS procedure not only achieves portal decompression (thus avoiding subsequent portal hypertension) but also improves stent inflow and reduces the risk of recurrent thrombosis. This is a rare report of rescuing a patient with fulminant liver failure secondary to BCS and PVMVT, without resorting to LT. In an era of severe organ shortage, we suggest that this technique could be considered as an alternative or bridge to transplantation in similar cases, or in those for whom transplant is contraindicated.

## Learning points

1. BCS and PVMVT is a rare and complex pathology with no standardized management algorithm.
2. TIPSS combined with pharmacomechanical thrombectomy of PV and SMV is seen as a useful “bridge” to LT or, in some cases, an alternative.
3. We would recommend that patients of this complexity are managed in a specialist centre under a multidisciplinary team experienced in LT.

## Consent

Written informed consent for the case to be published (including images, case history and data) was obtained from the patient for publication of this case report.
